# Healthcare professionals’ awareness of climate change and its association with eco-anxiety and environmental sensitivity: a cross-sectional study

**DOI:** 10.1186/s12913-026-15770-x

**Published:** 2026-10-01

**Authors:** Tuğba Özdemir, Tuğba Çetinol Taştemel, Gülendam Karadağ

**Affiliations:** 1 Department of Nursing, Faculty of Health Sciences, Istanbul Gedik University, Pendik, Istanbul, Türkiye; 2 Units Affiliated to the Rectorate, Çanakkale Onsekiz Mart University, Çanakkale, Türkiye; 3 Department of Public Health Nursing, Faculty of Nursing, Dokuz Eylul University, Izmir, Türkiye

**Keywords:** Climate change, Environmental sensitivity, Healthcare professionals, Eco-anxiety, Awareness

## Abstract

**Background:**

Healthcare professionals play an essential role in addressing the health impacts of climate change. Understanding how their awareness of climate issues and eco-anxiety relate to environmental sensitivity is critical for strengthening their capacity to respond to climate-related risks. This study aimed to examine the relationship between healthcare workers’ climate change awareness, levels of eco-anxiety, and their environmental sensitivity.

**Methods:**

A cross-sectional, correlational design was employed. The study included 460 healthcare professionals aged 18 years or older working in clinical settings. Data were gathered using a socio-demographic form, the Environmental Sensitivity Scale, the Global Climate Change Awareness Scale, and the Eco-Anxiety Scale. Analyses were conducted using SPSS 24.0, including descriptive statistics, Spearman correlation, and multiple linear regression.

**Results:**

Healthcare professionals demonstrated moderate levels of climate change awareness and environmental sensitivity, while eco-anxiety levels were moderate. Regression analysis showed that awareness of global climate change, being a nurse, being aged 45 years or older, and membership in an environmental organization were significant predictors of environmental sensitivity, whereas the rumination sub-dimension of eco-anxiety showed only a marginal, non-significant association (*p*=0.064).

**Conclusions:**

The findings indicate that cognitive engagement with climate-related concerns, climate awareness, and certain demographic characteristics are associated with environmental sensitivity among healthcare professionals. Enhancing educational efforts, institutional support, and environmental policies may strengthen their capacity to address climate-related health risks and promote environmentally responsible practices in healthcare settings.

**Supplementary Information:**

The online version contains supplementary material available at https://doi.org/10.1186/s12913-026-15770-x.

## Introduction

Climate change and the social and environmental health problems caused by it have emerged as an important public health problem at the global level. Extreme temperatures, air pollution, increased allergen levels, severe meteorological events, deterioration in water and food quality, forest fires, hurricanes and floods create multifaceted risks for human health through the direct and indirect pathways influenced by global climate change [1]. Climate change-driven heat waves have been linked to numerous negative health outcomes, including myocardial infarction, renal failure, and various mental health conditions [2]. Changing weather patterns undermine food security by diminishing agricultural productivity. Air pollution contributes to millions of premature deaths each year, primarily due to cardiovascular and respiratory diseases [3]. Furthermore, climate change and global warming exacerbate water scarcity and degrade water quality, intensifying competition for natural resources and potentially leading to forced migration and conflicts [4].

Climate change negatively affects both physical and mental health. Situations such as loss of livelihoods and uncertainty about the future negatively affect the mental well-being of individuals [5]. In addition to existing global problems such as pandemics, natural and human disasters, energy crisis and cost of living, extreme weather events and disruptions in the supply chain challenge health systems and create additional burdens [6]. The key objectives of the 2030 Sustainable Development Agenda play a vital role in reducing the impacts of climate change and advancing global efforts toward environmental protection. In achieving these goals, the awareness and sensitivity of healthcare professionals, who can create a positive impact in the society, gain importance [7].

Healthcare professionals’ awareness of climate change is critical in preventing environmental risks and fulfilling their advocacy roles effectively [8]. The environmental sensitivity of nurses directly affects the success of climate-based interventions for public health [9]. Previous research indicates that nurses often experience significant anxiety regarding climate change, yet simultaneously express a strong sense of hope and motivation to mitigate its impacts [10]. Several studies have shown that nurses may be inadequately informed about how their profession intersects with climate change issues, pointing to a clear need for focused educational support to enhance their awareness [11]. Studies examining nursing students’ understanding of environmental issues and their views on global climate change report that students tend to demonstrate substantial awareness. These findings also indicate that environmental health topics should be systematically integrated into nursing education and clinical in-service training to strengthen climate preparedness [1]. Taken together, existing studies have predominantly examined climate change awareness, eco-anxiety, or environmental sensitivity in isolation, or within single-discipline samples such as nursing students; none has jointly examined how awareness and eco-anxiety together relate to environmental sensitivity among practicing healthcare professionals across disciplines. Conceptually, this study draws on the premise that cognitive awareness of an environmental threat (climate change awareness) shapes affective responses to that threat (eco-anxiety), and that both awareness and affective engagement jointly influence behavioral and attitudinal orientation toward the environment (environmental sensitivity).

Climate change represents a substantial risk to population health, making it essential to develop strategies within the scope of health protection and health promotion to counter these impacts. Health professionals, who work in close contact with the public, play a key role in communicating strategies to address present and emerging health challenges arising from shifts in the global climate [12]. Although a growing body of evidence underscores the wide‑ranging health consequences associated with climate change, few studies have examined how health professionals’ level of climate awareness and their experiences related to eco‑anxiety shape their environmental sensitivity. To address the limited evidence in this area, the current research offers data derived from healthcare professionals and aims to inform initiatives that enhance their engagement in climate-related advocacy. This study investigates the link between healthcare workers’ knowledge of climate-related issues, their eco-anxiety, and their responsiveness to environmental matters.

## Research questions

### RQ1

What are the levels of health professionals’ familiarity with climate change, their experiences of eco-anxiety, and their degree of environmental sensitivity?

### RQ2

Do variations in health professionals’ climate-related awareness and their eco-anxiety experiences influence their environmental sensitivity?

H_1_: Healthcare professionals’ climate change awareness and eco-anxiety will significantly predict their environmental sensitivity.

## Method

### Design

A cross-sectional framework was utilized, allowing for the analysis of associations between key variables at a single point in time.

### Participants

The research population comprised healthcare professionals employed in a state hospital in western Türkiye, totaling 1,232 individuals. The minimum required sample size for this known population was calculated via OpenEpi version 3, using DEFF = 1, *p* = 0.50, d = 0.05, and a 95% confidence level. The calculation yielded a minimum sample size of approximately 293 participants. To improve statistical power and compensate for potential non-response, 460 healthcare professionals were ultimately enrolled in the study, having met the eligibility criteria and given their informed consent. Participants were recruited using a consecutive convenience sampling approach: data collection took place during mandatory in-service training sessions organized by the hospital administration, held every Wednesday between April and May 2025, and attended by healthcare professionals from the hospital’s 18 inpatient units and 9 intensive care units according to the hospital’s routine training schedule. The scheduling of these sessions was determined solely by the hospital administration and was independent of the research team. During each session, the researchers informed attendees about the purpose of the study and invited eligible healthcare professionals to participate. As data were collected from a single state hospital in western Türkiye, the findings may not be generalizable to healthcare professionals working in other regions or types of healthcare institutions.

### Inclusion criteria

Healthcare professionals aged 18 years or above who were engaged in clinical practice.

### Exclusion criteria

Individuals not working in healthcare roles and those who declined participation.

### Data collection place, methods and tools

Data were gathered between April and May 2025 through a self-administered questionnaire. The survey was administered in person, and the researcher was present to offer clarification when necessary. Data collection included a socio-demographic form and administration of the Environmental Sensitivity Scale, the Climate Change Awareness Scale, and the Eco-Anxiety Scale.

### Socio-demographic form

The instrument was constructed by the researchers with reference to relevant scholarly sources [1, 10, 13]. It includes a total of six items regarding gender, age, unit of employment, environmental education, membership in an environmental non-governmental organisation and income status of health professionals.

### Environmental sensitivity scale

The scale was created by Yeşil and Turan (2020). It contains 20 items structured under four sub-dimensions. The knowledge/emotion sub-dimension includes items G1, G3-G4, G6-G8, and G10-G11, Items were scored on a five-point Likert response format, with higher values indicating stronger agreement. The recycling sub-dimension includes D13-D16, the sensitive behaviour sub-dimension includes D12, D5, and D1, the caring behaviour sub-dimension includes D3, D7-D8-energy/product saving D9-D10. The scale yields scores ranging from a low of 20 to a high of 100. Higher scores reflect greater environmental sensitivity, whereas lower scores indicate weaker sensitivity. According to Yeşil and Turan (2020), the scale demonstrated a Cronbach’s alpha of 0.84. In the current study, the reliability of the scale, as measured by the internal consistency coefficient, was 0.86 [14].

### Global climate change awareness scale

The scale created by Deniz et al. (2021). It contains 21 items structured under four sub-dimensions. The Impacts on Natural and Human Environment sub-dimension includes items 1–9, the Awareness of Global Organisations and Agreements sub-dimension includes items 10–15, the Emerging Causes sub-dimension includes items 16–18, and the Energy Consumption Relationship sub-dimension includes items 19–21. The highest score of 105 and the lowest score of 21 can be obtained from the scale. An increase in the scale’s total score reflects a stronger understanding of global climate change. Cronbach’s alpha value for the reliability of the developed scale was calculated as 0.82 [15]. In this study, the internal consistency coefficient was found to be 0.95.

### Eco-Anxiety Scale

The scale created by Hogg et al. [16]. The scale was translated and culturally adapted for Turkish by Uzun et al. [17]. It contains 12 items structured under four sub-dimensions. The scale uses a 4-point Likert format, ranging from 0 (never) to 3 (always). It consists of Emotional Symptoms sub-dimension: items 1, 2, 3, and 4, Behavioural Symptoms: items 8, 9, and 10, Anxiety about Personal Impact: items 11, 12, and 13, and Recurrent Thought: items 5, 6, and 7. Higher scores on the scale reflect elevated levels of eco-anxiety. The total scale Cronbach’s alpha was 0.91 (Uzun et al., 2022), and in the present study, it was also 0.91.

### Analysing the data

Questionnaires with incomplete responses were excluded before analysis; the final analytic sample consisted of 460 fully completed questionnaires with no missing data. IBM SPSS 24.0 software was employed for data analysis. To describe the participants’ characteristics and the results of the scales, descriptive statistics were calculated, including counts, percentages, averages, and standard deviations. To determine whether the continuous variables followed a normal distribution, the Kolmogorov-Smirnov (KS) and Shapiro-Wilk tests were applied. Results indicated that the total scores of all three scales significantly deviated from a normal distribution (Eco-Anxiety Scale: KS(460)=0.085, *p*<0.001, Shapiro-Wilk=0.980, *p*<0.001; Global Climate Change Awareness Scale: KS(460)=0.074, *p*<0.001, Shapiro-Wilk=0.972, *p*<0.001; Environmental Sensitivity Scale: KS(460)=0.059, *p*=0.001, Shapiro-Wilk=0.986, *p*<0.001). Accordingly, non-parametric methods were used to explore associations among the study variables. Spearman’s rank correlation was used to explore associations among eco-anxiety, global climate change awareness, and environmental sensitivity. Multiple linear regression was subsequently performed to identify which variables independently predicted environmental sensitivity while controlling for the effects of other variables simultaneously, as this method allows for the estimation of each predictor’s unique contribution beyond its bivariate correlation. A multiple linear regression was carried out to explore which variables influence environmental sensitivity. The dependent variable in the model was environmental sensitivity, while rumination, awareness of global climate change, professional role, age, environmental education status, and environmental organization membership were treated as independent variables. The significance threshold was set at *p* < 0.05. To evaluate the adequacy of the regression model, post-hoc power calculations were carried out. Variance Inflation Factor (VIF) values were calculated for all predictors in the regression model to assess multicollinearity; all VIF values ranged from 1.03 to 1.14 (tolerance = 0.87–0.97), well below the conventional threshold of concern, confirming that the regression model was stable and not biased by multicollinearity. To evaluate the practical magnitude of each predictor’s contribution beyond statistical significance, Cohen’s f² effect size was calculated for each variable in the regression model, as well as for the overall model. No subgroup, interaction, or sensitivity analyses were performed in this study.

### Ethical considerations

The Ethics Committee Approval was obtained on 26.03.2025 with decision number: 05/08. Institutional permission was obtained from the Provincial Directorate of Health. Authorization to use the scales was granted via e-mail. Written informed consent was secured from all participants, all of whom willingly participated in the study. The data collection process was conducted in accordance with the principles of the Declaration of Helsinki.

## Findings

The majority of the health professionals included in the study were female, accounting for 74.6% of the sample, 57.4% were in the age range of 25–34 years, 71.5% were nurses, and 39.3% had an income level equivalent to expenditure. It was found that 53.5% of the participants did not receive any environmental education and 88.5% were not members of any environmental organisation (Table 1).

**Table 1:** Distribution of socio-demographic characteristics of the participants (*n* = 460)

	*n*	%
**Gender**		
Female	343	74.6
Male	117	25.4
**Age**		
18–24	12	2.6
25–34	264	57.4
35–44	140	30.4
45 and above	44	9.6
**Profession**		
Nurse	329	71.5
Other health professionals	131	28.5
**Income level income**		
Income exceeds expenses	41	8.9
Income equals expenditure	181	39.3
Less than their expenses	238	51.7
**Environmental education status**		
Yes	214	46.5
No	246	53.5
**Membership status in an environmental organization**		
Member	53	11.5
Not a member	407	88.5

The average overall score obtained on the Eco-Anxiety Scale was 13.75 ± 7.33, the mean total score of the Global Climate Change Awareness Scale was 74.29 ± 15.96 and the mean total score of the Environmental Sensitivity Scale was 61.78 ± 8.86 (Table 2).

**Table 2:** Mean scores of eco-anxiety, global climate change awareness scale and environmental sensitivity scales and subscales (*n* = 460)

	X ± SD.	Min.-Max.
**Eco-Anxiety Scale Total**	13.75 ± 7.33	0–39
Affective symptoms	4.67 ± 2.68	0–12
Behavioral symptoms	2.77 ± 2.20	0–9
Anxiety about personal impact	2.94 ± 2.04	0–9
Rumination	3.36 ± 2.15	0–9
**Global Climate Change Awareness Scale Total**	74.29 ± 15.96	21–105
Awareness of the Effects of Global Climate Change on the Natural and Social Environment	35.56 ± 6.58	9–45
Awareness of Global Organizations and Agreements	18.97 ± 6.59	6–30
Awareness of the Causes of Global Climate Change	9.08 ± 3.28	3–15
Awareness of the Energy Consumption Relation of Global Climate Change	10.67 ± 3.16	3–15
**Environmental Sensitivity Scale Total**	61.78 ± 8.86	32–91
Knowledge/ emotions	12.38 ± 5.17	8–40
Recycling	16.07 ± 3.20	4–20
Sensitive behavior	12.06 ± 1.95	3–15
Caring behaviour	21.25 ± 3.18	5–25

When the relationship between eco-anxiety, global climate change awareness, and environmental sensitivity was examined, a statistically significant, positive, and weak correlation was identified between the total mean score of the Environmental Sensitivity Scale and the Rumination sub-dimension of the Eco-Anxiety Scale (*p* < 0.05). It was found that there was a positive correlation of moderate strength between the total mean score of Global Climate Change Awareness and the total mean score of environmental sensitivity, as well as with the natural and social environment sub-dimension (*p* < 0.05). Weak, though statistically significant, positive correlations were also found with the awareness of global organisations and agreements sub-dimension, the emerging causes sub-dimension, and the energy consumption sub-dimension (*p* < 0.05) (Table 3).

**Table 3:** The relationship between environmental sensitivity scale and eco- anxiety and global climate change awareness scale

	Environmental Sensitivity Scale Total
**Eco-Anxiety Scale Total**	*r* = 0.082 *p* = 0.077
Affective symptoms	*r* = 0.078 *p* = 0.097
Behavioral symptoms	*r* = 0.027 *p* = 0.565
Anxiety about personal impact	*r* = 0.045 *p* = 0.335
Rumination	***r*** **= 0.147** ***p*** = 0.002*
**Global Climate Change Awareness Scale Total**	***r*** **= 0.343** ***p*** = 0.000**
Awareness of the Effects of Global Climate Change on the Natural and Social Environment	***r*** **= 0.328** ***p*** = 0.000**
Awareness of Global Organizations and Agreements	***r*** **= 0.287** ***p*** = 0.000**
Awareness of the Causes of Global Climate Change	***r*** **= 0.223** ***p*** = 0.000**
Awareness of the Energy Consumption Relation of Global Climate Change	***r*** **= 0.201** ***p*** = 0.000**

r=Spearman, *p < 0.05, **p < 0.001, *** Correlation coefficients less than 0.3 are interpreted as weak relationships

According to the results of the multiple linear regression analysis presented in Table 4, the significant predictors of environmental sensitivity were global climate change awareness, being a nurse, being 45 years old or older, and membership in an environment-related organisation. The rumination sub-dimension did not reach statistical significance (*p*=0.064) and showed a negligible effect size (f²=0.008), indicating only a marginal association with environmental sensitivity. The regression model was statistically significant (F = 38.574, *p* < 0.001), with an adjusted R² of 0.329, explaining 33.8% of the variance in environmental sensitivity. The 95% confidence intervals for each predictor confirmed the precision of these estimates: global climate change awareness (β = 0.154, 95% CI: 0.11–0.19), age 45 and above (β = 2.987, 95% CI: 0.69–5.28), being a nurse (β = 3.093, 95% CI: 1.59–4.59), and membership in an environmental organization (β = 10.486, 95% CI: 8.25–12.71) all showed confidence intervals that excluded zero, supporting the robustness of these associations. In contrast, the rumination sub-dimension showed a wide confidence interval that included zero (95% CI: -0.17-0.61), consistent with its non-significant p-value. Effect size analysis indicated a large overall model effect (f²=0.511); among individual predictors, membership in an environmental organization showed the largest unique effect (f²=0.189), while rumination showed a negligible effect (f²=0.008), consistent with its non-significant p-value (Table 4).

**Table 4:** Regression analysis on environmental sensitivity and descriptive characteristics

Variables	β	%95 CI	SE	t	*p*	f²	*R* ^2^	Adj. *R*^2^	F	*p*
Stable	45.181	41.86–48.49	1.686	26.790	**0.000**	-	0.338	0.329	38.574	0.000
Global Climate Change Awareness Scale Total	0.154	0.11–0.19	0.022	6.974	**0.000**	0.107				
Rumination Sub-scale	0.301	-0.17-0.61	0.162	1.858	0.064	0.008				
Age/45 and above	2.987	0.69–5.28	1.168	2.227	**0.011**	0.014				
Profession/Nursing	3.093	1.59–4.59	0.763	4.051	**0.000**	0.036				
Environmental education status/Yes	1.006	-0.42-2.42	0.726	1.387	0.166	0.004				
Membership status in an environmental organization/Member	10.486	8.25–12.71	1.134	9.245	**0.000**	0.189				

β, regression coefficient. SE, standard error, CI, 95% confidence interval, f², Cohen’s effect size for each predictor’s unique contribution (small ≥ 0.02, medium ≥ 0.15, large ≥ 0.35). Overall model effect size: f² = 0.511 (large)

A post-hoc power analysis for a regression model with six predictors (*n* = 460, f² = 0.511, α = 0.05) revealed that the statistical power of the test was approaching 1. This indicates that the model demonstrated a strong effect of the six predictors on the dependent variable, the sample size was more than sufficient to detect this effect, and the study’s statistical sensitivity was very high, ensuring the observed effect was reliably detected.

## Discussion

In this study, the association between healthcare professionals’ climate change awareness and eco-anxiety levels and their environmental sensitivity was examined. The findings revealed that healthcare professionals demonstrated moderate levels of climate change awareness and environmental sensitivity, while their eco-anxiety levels were at a moderate level. These findings indicate that healthcare professionals recognize climate change as an important issue, yet their level of anxiety remains moderate. H_1_ was partially supported; global climate change awareness significantly predicted environmental sensitivity, whereas overall eco-anxiety did not reach statistical significance, although its rumination sub-dimension showed a marginal association.

The findings of our study are consistent with results reported in previous research conducted with different populations. For instance, a study carried out with preschool teachers identified a positive and significant relationship between environmental sensitivity and environmentally conscious consumer behavior. As teachers’ levels of environmental sensitivity increased, their environmentally responsible consumption behaviors also improved, and conversely, heightened environmentally conscious consumption habits further strengthened their environmental sensitivity [18]. Similarly, a study involving teachers from various disciplines found that levels of environmental awareness, environmental sensitivity, and connectedness to nature were moderate, varied according to certain demographic characteristics, and were positively correlated with each other [19]. Additionally, studies involving adults have shown that those with higher environmental literacy tend to be more conscious of their ecological impact, which is associated with increased engagement in pro-environmental behaviors [20]. Collectively, these findings emphasize the critical role of environmental sensitivity in shaping positive environmental behaviors.

The analysis revealed that higher awareness of climate change among healthcare professionals was significantly associated with increased environmental sensitivity. This finding suggests that enhancing awareness may strengthen healthcare professionals’ pro-environmental behaviors. This result may be related to the role of awareness as a cognitive precursor to environmental engagement, whereby individuals who possess greater knowledge of climate-related risks may be more likely to recognize the personal and professional relevance of environmental issues, which in turn may heighten their attentiveness and responsiveness to environmental cues in daily practice. Fertelli (2023) reported that nurses’ climate change awareness was at a moderate level, while Orhan (2025) noted that although healthcare professionals demonstrated awareness of climate change, they were unable to advance this to higher levels [21, 22]. These findings indicate that awareness alone is not sufficient; in order for it to translate into concrete behaviors, it is necessary to develop environment-focused training programs and policies tailored to healthcare professionals.

In a study conducted by Tümer et al. (2024), the levels of awareness, anxiety, and hope regarding climate change among nursing students were examined [13]. The results showed that students’ awareness and anxiety about climate change positively influenced their hope for the prevention of climate change, suggesting that initiatives aimed at increasing awareness may motivate students to take action. Similarly, another study among nursing students reported that the majority had not received formal education on global climate change and environmental sensitivity, and that their knowledge was largely limited to information obtained through mass media [23]. Furthermore, a study investigating the attitudes of nursing academics toward climate change and environmental sensitivity found that although they possessed knowledge about climate change and incorporated certain behaviors and practices into their personal lives, they did not sufficiently emphasize the importance of this issue within their course curricula [24]. These findings collectively highlight that climate change and environmental sensitivity are not adequately integrated into current educational curricula, which may limit students’ knowledge and preparedness in this area.

According to the Intergovernmental Panel on Climate Change (IPCC), climate change is one of the most critical challenges of our time and has detrimental effects on human health. The consequences of climate change not only contribute to the development of physical health problems such as diabetes and cancer, but also negatively affect mental health by leading to conditions such as depression, anxiety, fear, and trauma [25–27]. Therefore, it is essential that all health professionals maintain a high level of awareness regarding climate change and its health impacts. In this context, the integration of environmental and climate change topics into educational curricula, along with the reinforcement of such knowledge through in-service training as part of professional development, is considered crucial for fostering environmental consciousness among health professionals and ensuring the sustainability of this awareness.

When examining the relationship between eco-anxiety and environmental sensitivity, it was found that the overall eco-anxiety score was not significantly associated with environmental sensitivity; the rumination sub-dimension showed a weak but statistically significant correlation with environmental sensitivity, although this did not translate into a statistically significant unique predictor when other variables were accounted for in the regression model. This result may be related to the repetitive and reflective nature of rumination, which represents a more cognitively engaged form of environmental concern than the broader eco-anxiety construct. The eco-anxiety construct additionally encompasses affective and behavioral symptoms that are less directly tied to deliberate reflection on environmental threats. The pathway from climate-related concern to environmental sensitivity may therefore operate primarily through sustained cognitive engagement rather than general emotional distress, consistent with cognitive theories of pro-environmental behavior suggesting that reflective processing, rather than affective arousal alone, more reliably translates concern into action-oriented attitudes.

In a study conducted by Baykara Mat and Yılmaz (2024), nursing students were found to have moderate levels of eco-anxiety, with the highest mean scores observed in the emotional symptoms sub-dimension [28]. This finding underscores that the impacts of climate change are experienced not only at a cognitive level but also on an emotional dimension. This highlights the need for psychosocial support and educational interventions that address both the emotional and cognitive aspects of climate change among healthcare students and professionals.

Seki Öz and Şen (2024) reported that health sciences students had moderate levels of eco-anxiety, but that this was not significantly associated with ecological footprint awareness [29]. Conversely, research with adult participants showed that those exhibiting greater sensitivity traits were more likely to develop a stronger environmental identity, experience elevated eco-anxiety, and engage in more pro-environmental behaviors [30]. Previous studies have also shown that as eco-anxiety levels increase, individuals engage in more intense reflections on climate change, a process that can negatively affect mental health [16, 31]. These findings suggest that while eco-anxiety may act as a motivating factor for raising awareness and encouraging pro-environmental behaviors, excessive levels may have detrimental consequences on psychological well-being. In this context, maintaining moderate levels of eco-anxiety among healthcare professionals is crucial, both for protecting their own mental health and for ensuring the sustainability of public health protection efforts. This study contributes to the limited body of research examining eco-anxiety, climate change awareness, and environmental sensitivity simultaneously among healthcare professionals in Turkey.

Türkiye’s strategic international significance, owing to its geographic location and climatic features shared with neighboring regions, lends broader relevance to the findings of this study beyond the national context, extending to neighboring countries and the global level. The awareness gaps and psychological responses identified in this study may be generalizable to healthcare professionals in neighboring regions exposed to similar climatic impacts. These findings may inform future research in climate-health among neighboring countries and globally, contribute to the shaping of climate-health education policies for healthcare professionals, and guide clinical practice initiatives aimed at enhancing environmental awareness. Accordingly, strengthening climate-health curricula and enhancing institutional support for healthcare professionals in Türkiye may also offer guidance for practices in regions with comparable geographic and climatic conditions.

## Conclusions

In this study, it was aimed to reveal the relationship between health care professional’s awareness levels of climate change, eco-anxiety levels and, environmental sensitivity. As a result of the study, it was determined that the awareness levels of healthcare professionals regarding climate change, their eco-anxiety levels, and their environmental sensitivity levels were all at a moderate level. The finding of a significant and positive relationship between climate change awareness and environmental sensitivity shows that the level of knowledge and awareness is associated with environmentally sensitive behaviours. Furthermore, while rumination showed a modest association with environmental sensitivity at the bivariate level, its contribution as an independent predictor did not reach statistical significance. In addition, it was revealed that health care professionals had intellectual awareness of environmental problems, but this did not turn into a high level of anxiety.

In line with these findings, it can be said that increasing the level of knowledge of healthcare professionals about climate change and environmental problems may be associated with stronger environmentally sensitive behaviours. Climate change and environmental health issues can be shared up-to-date by adding in-service training and awareness programmes for healthcare professionals. It may be beneficial to add relevant courses to the education curricula of healthcare professionals to increase awareness, raise the level of consciousness and support the transfer of information to the society. Individuals can be supported with supportive psychosocial interventions to cope positively with eco anxiety. In conclusion, this study provides a comprehensive understanding of the association between healthcare professionals’ climate change awareness and eco-anxiety experiences and environmental sensitivity, thereby filling an important gap in the literature. It also contributes to the development of strategies that integrate climate change education, psychosocial support, and environmental advocacy into healthcare practice.

## Limitations

This study was conducted with health professionals other than physicians, as the sample primarily comprised nurses, midwives, and allied health staff. Therefore, the findings may not be generalizable to physicians, whose professional roles and perspectives on climate change may differ from those of other healthcare professionals. The study was conducted in a single institution, which limits the generalizability of the findings, particularly given that the sample was predominantly nurses, female, and aged 25–34 years. Furthermore, as participants were recruited through a consecutive convenience sampling method based on attendance at scheduled training sessions, self-selection bias cannot be ruled out, as those who attended and volunteered may differ systematically from non-attendees. Future studies should consider multi-site designs and individual-level random sampling to improve generalizability. Additionally, as data were collected via self-administered questionnaires, responses may be subject to social desirability and self-report bias. The cross-sectional design also precludes any causal inference regarding the observed associations. Future longitudinal studies are needed to clarify the directionality of the relationships identified in this study.

## Supplementary Information

Below is the link to the electronic supplementary material.

Supplementary Material 1

Supplementary Material 2

## Data Availability

Data available on request from the authors.
