# Supplementary material for: Healthcare professionals’ awareness of climate change and its association with eco-anxiety and environmental sensitivity: a cross-sectional study
Source: BMC Health Serv Res. 2026 Oct 1;26:1373. doi: 10.1186/s12913-026-15770-x (PMC13651324; doi:10.1186/s12913-026-15770-x)
Supplement: Supplementary file 1 [file 12913_2026_15770_MOESM1_ESM.pdf]

Socio Demografic Form: The instrument was constructed by the researchers with reference to relevant scholarly sources (1–3). It includes a total of six items regarding gender, age, unit of employment, environmental education, membership in an environmental non-governmental organisation and income status of health professionals.

1. Please indicate your age.
   a) 18–24 b) 25–34 c) 35–44 d) 45 and above
2. Please indicate your gender.
   ( ) Female ( ) Male
3. Please specify your profession: …………….
4. Please indicate your income level.
   a) Income exceeds expenses
   b) Income equals expenses
   c) Income is lower than expenses
5. Have you received any education related to the environment?
   a) Yes b) No
6. Are you a member of any environmental organization?
   a) Yes b) No

4. Eco-Anxiety Scale: The scale created by Hogg et al. (4). The scale was translated and culturally adapted for Turkish by Uzun et al. (5). It contains 12 items structured under four sub-dimensions. The scale uses a 4-point Likert format, ranging from 0 (never) to 3 (always). It consists of Emotional Symptoms sub-dimension: items 1, 2, 3, and 4, Behavioural Symptoms: items 8, 9, and 10, Anxiety about Personal Impact: items 11, 12, and 13, and Recurrent Thought: items 5, 6, and 7. Higher scores on the scale reflect elevated levels of eco-anxiety. The total scale Cronbach's alpha was 0.91 (Uzun et al., 2022), and in the present study, it was also 0.91.

**Statements**	**Never**	**Sometimes**	**Often**	**Almost Always**
1. Feeling irritated, anxious, or tense				
2. Being unable to stop or control worrying				
3. Worrying excessively				
4. Feeling frightened				
5. Being unable to stop thinking about future climate change and other global environmental problems				
6. Being unable to stop thinking about past climate change-related events				
7. Being unable to stop thinking about environmental damage				
8. Having difficulty sleeping				
9. Having difficulty enjoying social settings with family and friends				
10. Having difficulty performing work and/or studying				
11. Feeling anxious about the impact of your personal behaviors on the world				

Statements	Never	Sometimes	Often	Almost Always
12. Feeling concerned about your personal responsibility/role in helping solve environmental problems				
13. Feeling concerned that your personal behaviors will contribute very little to solving the problem				

2. Environmental Sensitivity Scale: The scale was created by Yeşil and Turan (2020). It contains 20 items structured under four sub-dimensions. The knowledge/emotion sub-dimension includes items G1, G3-G4, G6-G8, and G10-G11, Items were scored on a five-point Likert response format, with higher values indicating stronger agreement. The recycling sub-dimension includes D13-D16, the sensitive behaviour sub-dimension includes D12, D5, and D1, the caring behaviour sub-dimension includes D3, D7-D8-energy/product saving D9-D10. The scale yields scores ranging from a low of 20 to a high of 100. Higher scores reflect greater environmental sensitivity, whereas lower scores indicate weaker sensitivity. According to Yeşil and Turan (2020), the scale demonstrated a Cronbach's alpha of 0.84. In the current study, the reliability of the scale, as measured by the internal consistency coefficient, was 0.86. (6).

Opinion Subdimension

Statements	Strongly Disagree	Disagree	Undecided	Agree	Strongly Agree
1. People littering the environment disturbs me.					
2. Unplanned urbanization disturbs me.					
3. Base stations located in residential areas negatively affect health.					
4. Unnecessary use of electricity disturbs me.					
5. I am disturbed by the lack of open green spaces in cities.					
6. Behaviors that harm green areas disturb me.					
7. Pollution of seas/natural water resources upsets me.					
8. The increasing warming of the Earth will cause disasters in the future.					

Behavioral Subdimension

Statements	Never	Rarely	Sometimes	Mostly	Always
9. I make efforts to inform people about air pollution.					
10. I pay attention not to litter.					
11. I make an effort to take opportunities for planting trees.					
12. I pay attention to saving water.					
13. I avoid making noise at levels that may disturb others.					
14. I try to use both sides of paper.					
15. I always try to economize on disposable paper products (napkins, wet wipes, toilet paper, etc.).					
16. After using electronic devices such as TVs and computers, I do not leave them on standby mode; I turn them off.					
17. I try to be a role model regarding environmental awareness.					
18. I make sure not to dispose of used batteries with household waste and instead place them in battery recycling bins.					
19. I separate waste and dispose of it in appropriate recycling bins.					
20. I prefer products with recyclable packaging.					

3. Global Climate Change Awareness Scale: The scale created by Deniz et al. (2021). It contains 21 items structured under four sub-dimensions. The Impacts on Natural and Human Environment sub-dimension includes items 1-9, the Awareness of Global Organisations and Agreements sub-dimension includes items 10-15, the Emerging Causes sub-dimension includes items 16-18, and the Energy Consumption Relationship sub-dimension includes items 19-21. The highest score of 105 and the lowest score of 21 can be obtained from the scale. An increase in the scale's total score reflects a stronger understanding of global climate change. Cronbach's alpha value for the reliability of the developed scale was calculated as 0.82 (7). In this study, the internal consistency coefficient was found to be .95.

Not Aware at All (1), (2), (3), (4), (5) Completely Aware

Statement	1	2	3	4	5
1. Migration routes and habitats of birds are changing along with global warming.					
2. In parallel with global climate change and global warming, some natural monuments, natural areas, and national parks may disappear.					
3. Global climate change will further accelerate the extinction of plant and animal species.					

Statement	1	2	3	4	5
4. The spread of tropical plants and fish that prefer warm climates and waters toward the poles is an indicator of global climate change.					
5. Due to global climate change, the habitats of species such as polar bears are shrinking.					
6. Traditional agricultural methods are becoming more difficult because of global climate change.					
7. Some cases of mass migration are significant consequences of global climate change.					
8. Changes will occur in some winter tourism centers due to global warming.					
9. Types of tourism activities in certain regions may change due to global warming.					
10. The Kyoto Protocol is the only framework convention supporting the fight against global warming and climate change at the international level.					
11. The Montreal Protocol is a framework aimed at protecting the ozone layer.					
12. In 1988, the Intergovernmental Panel on Climate Change (IPCC) was established to assess the risks of climate change caused by human activities.					
13. Since 1968, the Club of Rome has been preparing reports highlighting threats related to economy, politics, the environment, and global climate change.					
14. International regulations and quotas on the use of CFCs (chlorofluorocarbons) were introduced through the Montreal Protocol.					
15. Under the Paris Agreement signed in 2016 regarding global climate change, it was agreed that the global average temperature increase should be limited to 1.5–2.0°C.					
16. Expansion of rice cultivation areas is among the agricultural activities contributing to global warming.					
17. Increased cattle breeding contributes to global warming.					
18. Consuming more meat increases global warming.					
19. The more non-renewable energy we consume, the more carbon dioxide emissions increase.					
20. Increased use of energy-saving devices reduces risks arising from global climate change.					
21. Using solar energy instead of fossil fuels is one of the measures that can be taken against global climate change.					

References

1. Duran S, Kaynak S. Climate change worry among nurses and their hope levels for climate change prevention. BMC Nurs [Internet]. 2024 Jun 21;23(1):419. Available from: https://bmcnurs.biomedcentral.com/articles/10.1186/s12912-024-02067-9

2. Incesu O, Yas MA. The relationship between nursing students' environmental literacy and awareness of Global Climate Change. Public Health Nurs [Internet]. 2024 Jan 24;41(1):67–76. Available from: https://onlinelibrary.wiley.com/doi/10.1111/phn.13255

3. Tümer A, İpek M, Ercan Z. Nursing Students' Awareness, Worry and Hope Levels Regarding Climate Change: A Cross-Sectional and Correlational Study. J Public Heal Nurs. 2024;6(1):29–38.

4. Hogg TL, Stanley SK, O'Brien L V., Wilson MS, Watsford CR. The Hogg Eco-Anxiety Scale: Development and validation of a multidimensional scale. Glob Environ Chang [Internet]. 2021 Nov;71(March):102391. Available from: https://linkinghub.elsevier.com/retrieve/pii/S0959378021001709

5. Uzun K, Ozturk AF, Karaman M, Cebecı F, Altın MO, Arıcı A, et al. Adaptation of the Eco-Anxiety Scale to Turkish: A Validity and Reliability Study. Arch Heal Sci Res [Internet]. 2022 Jun 7;9(2):110–5. Available from: https://archhealthscires.org/en/adaptation-of-the-eco-anxiety-scale-to-turkish-a-validity-and-reliability-study-13379

6. Yeşil M, Turan Y. A Scale Development Study on Environmental Sensitivity. Ordu Univ Soc Sci Inst Soc Sci Res J. 2020;10(2):418–35.

7. Deniz M, İnel Y, Sezer A. Awareness Scale of University Students About Global Climate Change. Int J Geogr Geogr Educ (IGGE). 2021;(43):252–64.
